# Gambling disorder risk factors in a population of online sports betting players in Sfax

**DOI:** 10.1192/j.eurpsy.2022.2127

**Published:** 2022-09-01

**Authors:** A.S. Ellouze, J. Ben Thabet, M. Maalej, R. Feki, I. Gassara, N. Smaoui, S. Omri, L. Zouari, N. Charfi, M. Maalej

**Affiliations:** Hedi Chaker University Hospital, Psychiatry C, Sfax, Tunisia

**Keywords:** risk factors, Gambling Disorder, Impulsivity, Stress

## Abstract

**Introduction:**

Online sports betting (OSB) is frequently associated with gambling disorder (GD). In Tunisia, no study on this has been done so far.

**Objectives:**

To detect GD in a population of Tunisian OSB players, and to identify its risk factors.

**Methods:**

This was a cross-sectional study of 58 male OSB players in the city of Sfax. The GD was assessed by a questionnaire relating to the DSM-V criteria. Depression, anxiety and stress were assessed using the DASS scale, gambling motivations using the GMQ-F scale, and impulsivity using the UPPS-P scale.

**Results:**

The mean age was 37.4 ± 8.29 years. The prevalence of JAP was 53.4%. On univariate analysis, the factors associated with GD were university level of education, the practice of other gambling, daily gambling, gambling spending > 300 Dinars / month , gambling duration > 3 years, the frequency of winning >1 win /6months , the occurrence of a Big Win, total GMQ-F score, coping motivation , and financial motivation. In the multivariate study, GD risk factors were gambling spending > 300 Dinars / month (p = 0.011; ORa = 223.16), financial motivation (p = 0.022; ORa = 3.967), pathological stress (p = 0.036; ORa = 224.388) and inversely associated with the age at onset of gambling (p = 0.026; ORa = 0.751) and the UPPS score (p = 0.011; ORa = 0.6).

**Conclusions:**

Our results push us to deepen our knowledge and our studies concerning this problem in our country and to reflect on the management and prevention measures.

**Disclosure:**

No significant relationships.

